# Machine learning empowered formulation design, optimization and characterization of nanoparticulate drug delivery systems: Current applications, challenges, and future perspectives

**DOI:** 10.1016/j.apsb.2025.12.011

**Published:** 2025-12-10

**Authors:** Chunyan Shen, Mengyan Zhang, Meiting Lu, Errong Chang, Ziting Gao, Weikang Ban, Qiang Liu, Zhong Zuo, Cuiping Jiang

**Affiliations:** a Guangdong Provincial Key Laboratory of Chinese Medicine Pharmaceutics, School of Traditional Chinese Medicine, Southern Medical University, Guangzhou 510515, China; b Guangdong Basic Research Center of Excellence for Integrated Traditional and Western Medicine for Qingzhi Diseases, Guangzhou 510515, China; c Guangdong Provincial People's Hospital (Guangdong Academy of Medical Sciences), Southern Medical University, Guangzhou 510515, China; d School of Pharmacy, Faculty of Medicine, The Chinese University of Hong Kong, Hong Kong, China

**Keywords:** Artificial intelligence, Machine learning, Nanoparticulate drug delivery system, Formulation development, Formulation design and optimization, Nanomedicine, Polymeric nanoparticles, Lipid nanoparticles

## Abstract

Nanoparticulate drug delivery systems (NDDS) have revolutionized modern medicine by significantly improving drug targeting, bioavailability, and therapeutic efficacy. Despite the clinical success of over 90 approved nanomedicines, the development of NDDS remains challenging due to the complexity of formulation design, optimization, and characterization processes. Artificial intelligence, particularly machine learning (ML), offers powerful data analytics and predictive capabilities that can address these challenges. This review systematically summarizes recent advances in ML applications across various NDDS formulations, including polymeric nanoparticles, lipid nanoparticles, liposomes, solid lipid nanoparticles, nanostructured lipid carriers, nanoemulsions, nanosuspensions, lipid-based hybrid NDDS, self-emulsifying drug delivery systems, niosomes, and nanocrystals. We also summarize how ML algorithms could help predict critical quality attributes of NDDS, such as particle size, shape, surface properties, drug encapsulation efficiency, drug loading efficiency, drug release behavior, and stability. Furthermore, we discuss existing challenges and prospects for the formulation development empowered by ML in NDDS. In conclusion, this review provides a comprehensive overview of the transformative potential of ML in improving the formulation development of nanomedicines, ultimately accelerating their clinical translation.

## Graphical abstract

This review updates recent advances in ML applications for diverse nanoparticulate drug delivery systems formulation development, covering design, optimization, and characterization.

**Figure:**
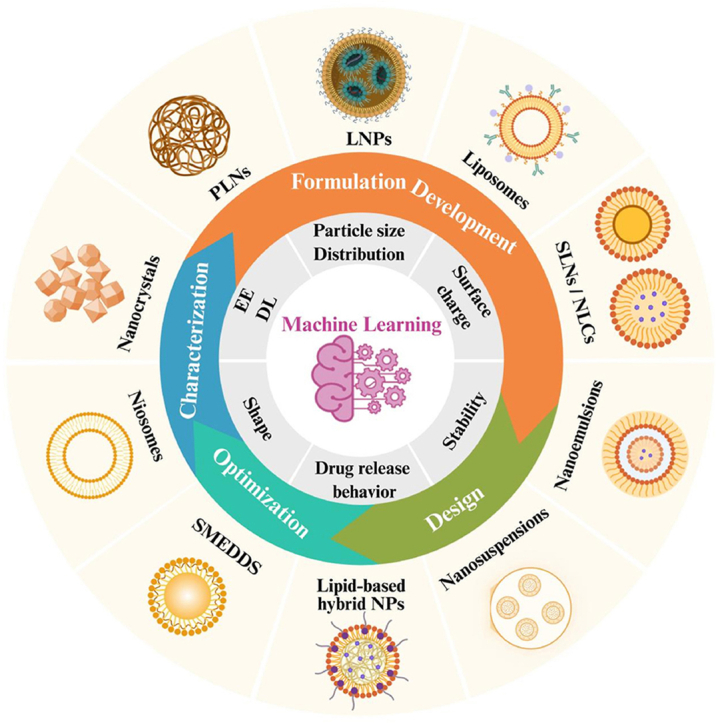

## 1 Introduction

Nanoparticulate drug delivery systems (NDDS) represent a revolutionary breakthrough in modern drug delivery technology. These systems employ carriers ranging in size from 1 to 1000 nm to precisely deliver drugs to target tissues or cells, thereby significantly enhancing the targeting ability, bioavailability, and therapeutic efficacy of the drugs^1^. In recent years, various nanoparticulate platforms, including liposomes, polymeric nanoparticles, and nanoemulsions, have been widely utilized in the field of drug delivery^2^. To date, over 90 nanomedicines have been approved for marketing globally, which fully demonstrates the immense clinical value of NDDS^3^. Nevertheless, the development of NDDS is accompanied by numerous challenges. The uncertain structure–function relationships impede the rational design of NDDS for targeted therapeutic outcomes. Besides, the optimization of the formulation and manufacturing process is complex, requiring the careful selection and fine-tuning of various components and preparation techniques such as emulsification, solvent evaporation, and nanoprecipitation, each with its specific applicable conditions and limitations. Moreover, the physicochemical heterogeneities inherent in these systems make their precise characterization particularly difficult^4^. Regardless, all the aforementioned formulation-related technical and characterization difficulties during NDDS development often led to a heavy reliance on traditional trial-and-error approaches, which consequently imposed substantial costs and time demands. Given these significant obstacles, there is a pressing need for innovative strategies to overcome these limitations^5^.

Faced with these challenges, the application of artificial intelligence (AI) technology offers new perspectives and methodologies for the development of NDDS^6^^,^^7^. Machine learning (ML) is an application of AI that leverages statistical methods to uncover patterns in diverse data forms, including text, images, or any digitally stored information^8^. It focuses on enabling machines to learn and improve from experience, essentially using computational models to extract information and make predictions based on those patterns without being explicitly programmed^9^, ^10^, ^11^. In the development of NDDS, the robust data processing and predictive capabilities of ML algorithms are reshaping the paradigms of formulation design, optimization, and characterization^12^^,^^13^. For instance, ML can screen appropriate carrier materials and drugs by constructing quantitative structure–property relationship (QSPR) models for the design of NDDS^14^^,^^15^. By analyzing vast amounts of experimental data, ML is capable of establishing correlation models between the formulation and process information of NDDS and their physicochemical properties, accelerating their optimization^4^. Additionally, ML algorithms can assist in characterizing nanoparticles (NPs) by analyzing their physicochemical properties, such as size, distribution^16^, ^17^, ^18^, and morphological structural features^19^, ^20^, ^21^, ^22^, ^23^.

Although there are a few recent reports on the application of ML in the formulation development of liposomes^24^ or lipid nanoparticles (LNPs)^25^, a systematic review of ML utilization in the formulation development of diverse NDDS remains unavailable. Our current review aimed to update recent advances of ML application in the development of a broader range of NDDS, including not only liposomes and LNPs, but also polymeric nanoparticles (PLNs), solid lipid nanoparticles (SLNs), nanostructured lipid carriers (NLCs), nanoemulsions, nanosuspensions, lipid-based hybrid NDDS, self-emulsifying drug delivery systems (SMEDDS), niosomes, and nanocrystals. In addition, we summarize how ML is applied to formulation design, optimization, and characterization, highlighting ML-aided critical metrics predictions, such as particle size/distribution, shape, surface properties, encapsulation efficiency (EE), drug loading (DL), drug release behavior, and stability. Furthermore, this review discusses the existing challenges and prospects of AI-driven formulation development of NDDS, aiming to promote further clinical applications of nanomedicine technology.

**Figure 1:**
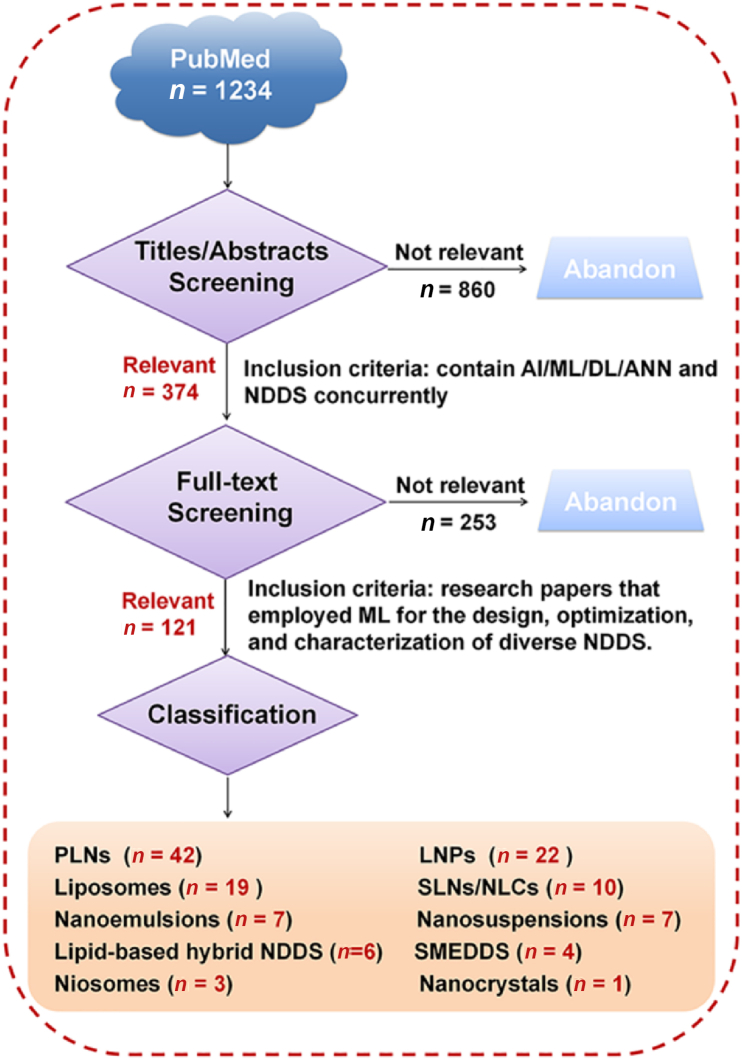
Flowchart of literature screening on ML empowered NDDS formulation development.

To identify relevant literature, a systematic search was conducted on PubMed for publications within the last 15 years. The search terms employed were: (((ai artificial intelligence [MeSH Terms]) OR (machine learning [MeSH Terms]) OR (deep learning [MeSH Terms]) OR (artificial neural network [MeSH Terms])) AND ((nanoparticles [MeSH Terms]) OR (drug delivery systems [MeSH Terms]) OR (nanomedicine [MeSH Terms]))) AND (“2010/01/01” [Date-Publication]: “2025/03/27” [Date-Publication]). As illustrated in Fig. 1, titles and abstracts of articles from PubMed were initially screened for their relevance to AI/ML/deep learning/ANN and NDDS. The eligible articles with full text that included the application of ML techniques in the design, preparation, or characterization of NDDS were eventually selected for the current review.

## 2 Overview of commonly used ML tools for NDDS formulation development

As one of the most prominent subsets of AI, ML encompasses major branches such as deep learning and artificial neural networks (ANN)^26^, and enables systems to learn and improve from experience automatically^27^. ML consists of four major procedures, including variable input, feature engineering, model training, and result outputs^28^^,^^29^. Based on the learning approach, ML can be classified into supervised learning, unsupervised learning, semi-supervised learning, and reinforcement learning^30^.

In the context of NDDS formulation development, supervised learning stands out as the most utilized ML technique. It is‌ a task-driven ML approach whose core process involves training mathematical models using labeled datasets and employing the trained models to predict outputs for new data^31^. Such a method is commonly applied to address two types of problems: ‌classification‌ and ‌regression‌. In classification tasks, models are trained to assign new variables to predefined categories based on labeled training data. The performance of classification models is typically assessed by accuracy, precision, recall, F1 score, area under the receiver operating characteristic curve, and confusion matrices. For regression tasks, algorithms aim to estimate associations between different variables by predicting continuous outcomes. Their performance is typically evaluated using mean squared error (MSE)‌, root mean squared error (RMSE), mean absolute error (MAE)‌, and coefficient of determination (*R*^2^)^32^‌. Based on their underlying principles, supervised learning algorithms can be broadly categorized into several distinct groups: linear models (*e.g.*, linear regression, multiple linear regression (MLR), logistic regression), tree-based models (*e.g.*, decision tree (DT), random forest (RF), eXtreme gradient boosting (XGBoost), light gradient boosting machine (LightGBM), least squares boosting (LSBoost)), kernel-based methods (*e.g.*, support vector machine (SVM), gaussian processes (GP)), instance-based methods (*e.g.*, k-nearest neighbor (KNN)), and neural network-based models (*e.g.*, artificial neural network (ANN), multilayer perceptron (MLP), deep neural network (DNN)). Detailed descriptions of these specific algorithms are well-documented in numerous reviews^28^^,^^33^, ^34^, ^35^ and briefly summarized in Table 1. Moreover, genetic algorithms (GA) and Bayesian optimization (BO) are commonly used to enhance ML algorithms by optimizing hyperparameters, with GA broadly exploring potential settings through evolutionary mimicry and BO more efficiently selecting subsequent settings *via* a predictive model^36^^,^^37^.

**Table 1:** Summaries of commonly used supervised learning tools for NDDS formulation development.

Type	ML tools	Description	Advantages	Disadvantages	Task
Linear	Linear regression	A linear model for predicting continuous values by fitting a line to minimize prediction errors.	Easy to understand, implement, and interpret, fast training and prediction speed.	Only suitable for linear relationships.	Regression
	MLR	A statistical method that models the linear relationship between a dependent variable and two or more independent variables.	Easy to understand and implement, capable of handling multiple variables.	Sensitivity to outliers, poor at capturing nonlinearity.	Regression
	Logistic regression	A linear model for binary classification that estimates probabilities using a logistic function to make predictions.	Simple, interpretable, and efficient in handling linear problems.	Only suitable for linear modeling, sensitive to feature scaling issues.	Classification
Tree-based	DT	A model based on a hierarchical tree structure, designed to make predictions *via* recursive data partitioning.	Easy to understand, interpret, and manage non-linearities, robust against noise.	Unstable, prone to overfitting. Not suitable for big datasets.	Classification regression
	RF	An ensemble of decision trees that uses voting or averaging for large datasets.	Analogous to decision trees, offers resistant to overfitting.	Complex, long training time, poor interpretability.	Classification regression
	XGBoost	An ensemble learning algorithm based on gradient boosting that optimizes model efficiency and performance.	Efficient for large-scale data, high prediction accuracy, offers resistant to overfitting.	Complex parameter tuning, sensitive to outliers, poor interpretability.	Classification regression
	LightGBM	An efficient ensemble gradient boosting framework based on decision trees for large datasets.	Fast training, high efficiency, supports distributed computing.	Complex, poor interpretability, prone to overfitting.	Classification regression
	LSBoost	An ensemble learning algorithm that combines multiple weak regression models to create a stronger predictive model.	High accuracy, simple and easy to implement, robust to outliers.	Sensitive to noisy data, outliers, and overfitting.	Regression
Kernel-based	SVM	An algorithm that separates different classes of data by finding the optimal decision boundary with the maximum margin for small datasets.	Handles high-dimensional data, generalizes well, robust to outliers, resists overfitting.	Sensitive to parameters, long training, poor interpretability, not for large datasets.	Classification regression
	GP	A non-parametric Bayesian model that provides uncertainty estimates for predictions.	Provides uncertainty estimates, interpretable for small to medium datasets.	High computational cost, sensitive to hyperparameters, slow training time.	Classification regression
Instance-based	KNN	An algorithm that finds the K closest neighbors to a new data point and uses their outputs to determine its prediction.	Simple and easy to implement, robust to outliers, suitable for small datasets.	Complex, poor interpretability, intolerant of noise.	Classification regression
Neural network-based	ANN	A neuron-mimicking network models learn data patterns from large datasets by adjusting connection weights.	Capable of complex non-linear relationships, strong generalization and adaptive learning abilities.	High data demand, slow training, complex tuning, poor interpretability.	Classification regression
	MLP	A feedforward neural network that learns complex patterns through multiple layers of neurons	Capable of complex non-linear relationships, strong generalization.	Prone to overfitting, high data demand, complex tuning, poor interpretability.	Classification regression
	DNN	A neural network with multiple layers, incorporating feedback and feedforward loops.	Capable of complex non-linear relationships, excels in complex tasks like image, speech, and text.	High data demand, slow training, complex tuning, poor interpretability.	Classification regression

MLR, multiple linear regression; DT, decision tree; RF, random forest (RF); XGBoost, eXtreme gradient boosting; LightGBM, light gradient boosting machine; LSBoost, least squares boosting; SVM, support vector machine; GP, gaussian processes; KNN, k-nearest neighbor; ANN, artificial neural network; MLP, multilayer perceptron; DNN, deep neural network.

## 3 Application of ML approach in NDDS formulation development

Traditionally, developing NDDS has often been a slow and expensive process reliant on time-consuming trial-and-error experimentation. Recognizing the powerful data processing and predictive capabilities of ML, researchers are increasingly turning to ML algorithms. These algorithms are now being leveraged to assist in the design, optimization and characterization of NDDS formulations, thereby accelerating the overall development cycle. Based on the number of retrievals for different NDDS shown in Fig. 1, the following section summarizes and discusses the applications of ML in the development of various NDDS in the following order: PLNs, LNPs, liposomes, SLNs, NLCs, nanoemulsions, nanosuspensions, lipid-based hybrid NPs, SMEDDS, niosomes, and nanocrystals.

### 3.1 Polymeric nanoparticles (PLNs)

PLNs are typically prepared from natural or synthetic polymers using physical or chemical methods^38^. They can achieve controlled drug release and targeted delivery through the degradation of the polymer matrix and surface modifications. Based on the origin of the polymeric materials, PLNs are classified into synthetic polymer-based PLNs and natural biopolymer-based PLNs as described below^39^.

#### 3.1.1 Synthetic polymer-based PLNs

Among synthetic polymers, poly(lactic-*co*-glycolic acid) (PLGA) has gained significant attention in drug delivery due to its outstanding biodegradability, biocompatibility, and controlled drug release behavior, making it a preferred nanocarrier^40^. Nanoprecipitation is a popular and scalable method for the preparation of PLGA-based PLNs (termed PLGA-NPs), involving rapid mixing of an organic PLGA phase with an aqueous surfactant solution to induce NPs formation *via* solvent diffusion and reduced polymer solubility^41^. The size of PLGA-NPs in nanoprecipitation is critically influenced by factors such as the types and concentrations of anti-solvents (*e.g.*, surfactants) and the polymer itself^42^. Building on this understanding, Seegobin et al.^43^ developed five supervised ML models (*i.e.*, XGBoost, RF, KNN, SVM, MLP) to predict the size and zeta potential of PLGA-NPs using the types and concentrations of PLGA and anti-solvent as inputs. It was found that XGBoost outperformed other models in predicting particle size but showed higher errors for zeta potential (Table 2^43-74^). The dichotomy between size and zeta potential predictability motivated Huwaimel et al.^44^ to pioneer a hybrid framework integrating advanced preprocessing (*e.g.*, isolation forest outlier detection, min-max normalization, one-hot encoding) with ensemble learning. Comparative *R*^2^ analysis revealed that bagging-enhanced support vector regression (BAG-SVR) achieved unprecedented accuracy for particle size prediction, while bagging-enhanced polynomial regression (BAG-PR) excelled in zeta potential modeling, thereby addressing the limitations of single-algorithm models like XGBoost^43^. Capitalizing on these methodological advancements, Almansour and colleagues^45^ further refined the paradigm by incorporating advanced preprocessing strategies (*i.e.*, leave-one-out encoding and local outlier factor) alongside the Bat optimization algorithm to enhance Bagging and adaptive Boosting-based KNN (*i.e.*, Bag-KNN, ADA-KNN), and Bat-optimized small-size KNN regression (SBNNR) models. Results demonstrated that ADA-KNN outperformed other models in particle size prediction, while SBNNR achieved exceptional accuracy for zeta potential by integrating generative adversarial networks and deep feature extraction to address data sparsity. Collectively, these studies highlight the key role of integrating advanced preprocessing, algorithmic optimization, and ensemble techniques to enhance the predictive efficacy of ML models in PLNs formulation optimization.

**Table 2:** Summary of ML tools used in the formulation development of PLNs based on synthetic polymer and natural biopolymer.

Type of polymer	Type of PLNs	Preparation method	Main ML tool	Input feature	Output feature	Ref.
Synthetic polymer	PLGA-NPs	Nanoprecipitation	XGBoost	PLGA type/conc., anti-solvent type/conc.	Size, zeta potential	43
			BAG-SVR, BAG-PR	PLGA type/conc., anti-solvent type/conc.	Size, zeta potential	44
			ADA-KNN, SBNNR	PLGA type/conc., anti-solvent type/conc.	Size, zeta potential	45
		Modified supercritical antisolvent technique	ANN	Solution flow rate, ratio between washing time and injection time, ultrasonic power, molar ratio between CO_2_ and solution, static agitation time	Size, yield, DL	46
		Electrospray	ANN	Voltage, internal diameter of needle (nozzle), polymer: protein ratio	Size, distribution	47
		Double emulsion method	GP	Drug log*P*, PLGA Mw, PLGA LA/GA ratio, PLGA: drug ratio	EE, IC_50_	48
		Microfluidics	DT, TGAN	LA/GA, drug 1/2, drug 1/2 conc., method, surfactant, solvent 1/2, PVA conc./Mw, PLGA 1/2 conc./Mw, surfactant conc., PEG Mw, flow ratio, aqueous flow, inner diameter, total flow	Size	49
		Nanoprecipitation or double emulsion, microfluidics	SVR, linear regression	LA/GA ratio, PLGA Mw, PEG layer, synthesis methods, solvents, surfactants	Size, EE, DL	50
		Not mentioned	GP	Size	EE, DL	51
	PCL-NPs	Nanoprecipitation	Neuro-fuzzy logic	Stabilizer type/conc., the solvent/antisolvent ratio, solvent volume, the injection inner diameter (IID), mixing time, linear flow rate, the percentage of acetone, acetonitrile, or methanol in the solvent phase, PCL Mw/conc.	Size, PDI, %Peak 1, %Peak 2, %Pd Peak 1, and number of peaks	52
	Polymethacrylate-NPs	Nanoprecipitation	ANN, neurofuzzy logic	Acetone content, ERS conc., P188 conc., total volume, solvent: antisolvent ratio, agitation type/intensity, temperature, injection inner diameter, linear flow rate, mixing time, flux type	Size, PDI, zeta potential, number of particle populations	53
		Nanoprecipitation	GCN-FCNN^a^	Degree of polymerization, nanoprecipitation parameters	Size	54
	Polydimethylsiloxane-NPs	Nanoprecipitation	MLP	Surfactant conc., polymer conc., storage temperature	Size, PDI	55
	PHBV-NPs	Emulsification and solvent evaporation technique	ANN	Time	Released drug	56
	PLGA, PVA, PCL, EC-NPs	Emulsification solvent evaporation method	ANN	Polymer viscosity, contact angle and interfacial tension	Size, PDI	57
	PEG-PLA-NPs	Nanoprecipitation	ANN	Polymer Mw, polymer: drug ratio, number of blocks	Size, EE	58
	PLA-PEG-PLA-NPs	Nanoprecipitation	ANN	Polymer conc., amount of drug, solvent ratio and mixing rate	Size	59
	Poly(beta-amino ester)-NPs	Not mentioned	RF	Polymer properties	Transfection efficiency	60
	Antibiotics-derived polymers-NPs	Not mentioned	SVR	Polymer properties	Transfection efficiency	61
	Poly(allylmethacrylamide)-NPs	Not mentioned	BO, SHAP	Polymer properties	Transfection efficiency	62
Natural polymer	Chitosan-NPs	Ionotropic gelation	ANN	Cs conc., TPP conc., Cs: TPP mass ratio	Size, zeta potential, yield%	65
			ANN	PEG chain length, chitosan/PEG ratio, pH of solution	Size, zeta potential	67
		Polyelectrolyte complexation	ANN	Chitosan and albumin conc., pH, and reaction time	Size, DL, cytotoxicity	68
		Ultrasonication	ANN	Chitosan conc., buffer pH, amplitude and time of sonication	Size	69
		FNP	Bagging	The numbers of reactive group and monomer in cationic/anionic polymer, the conc. of cationic/anionic polymer, flow speed	Size, PDI	70
	Agar nanospheres	Natural polymer coacervation	ANN, GA	CaCl2 conc., agar conc., HPβCD conc., homogenizer speed	Size, PDI, zeta potential, DL, drug release	71
	Fucoidan-NPs	Electrostatically driven complexation	ANN-DOE	Fucoidan: polyethyleneimine ratio	Size, EE	72
	Gelatin-NPs	Nanoprecipitation and two-step desolvation	ANN, MLR	Gelatin conc., glutaraldehyde conc., poloxamer 407 conc.	Size, EE	73
	HA-derivative NPs	Microfluidics	ANN	Flow rates, polymer conc.	Size	74

a GCN, graph convolutional network; FCNN, fully connected neuronal network.

**Figure 2:**
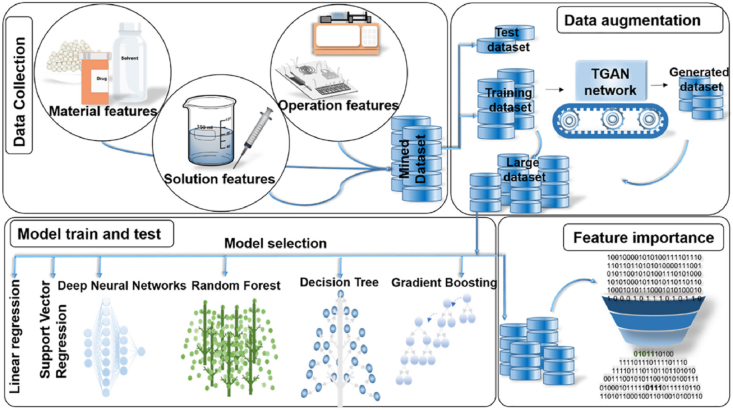
Workflow for ML-aided formulation optimization of PLGA-NPs synthesized *via* microfluidics. Reprinted with the permission from Ref. 49. Copyright © 2025 American Chemical Society.

Beyond optimizing PLGA-NPs prepared *via* nanoprecipitation, multiple ML techniques, including linear regression, ANN, GP, SVR, and DT, have been employed to accelerate the formulation and process optimization of PLGA-NPs through methods such as the modified supercritical antisolvent technique^46^, electrospraying^47^, double emulsion^48^, microfluidics methods^49^^,^^50^, and etc.^51^ (Table 2). Besides treating physicochemical parameters (*e.g.*, size, zeta potential, PDI, drug encapsulation efficiency (EE), drug loading efficiency (DL)) as output parameters, therapeutic outcome was also regarded as an output indicator. For example, Dong et al.^48^ developed GP algorithms to reveal the quantitative relationships between formulation variables and IC_50_ in ovarian cancer cells for double-emulsion-derived PLGA NPs. To address data scarcity, Mihandoost and coworkers^49^ innovatively constructed a DT algorithm with a tabular generative adversarial network (TGAN) to augment experimental datasets by synthetically generating training and testing subsets (Fig. 2)^49^. This approach aided in uncovering the factors affecting the particle size of PLGA-NPs synthesized *via* microfluidics. Feature importance analysis revealed that the concentration of poly(vinyl alcohol) (PVA) and the ratio of lactide to glycolide (LA/GA) in the PLGA copolymer are the main determinants.

In addition to PLGA-NPs formulation, ML has aided optimization of other PLNs, including polycaprolactone (PCL)-NPs^52^, polymethacrylate-NPs^53^^,^^54^, polydimethylsiloxane-NPs^55^, and poly(3-hydroxybutyrate-*co*-3-hydroxyvalerate) (PHBV)-NPs^56^, as summarized in Table 2. Among the ML tools used, ANN is the most widely used model^53^^,^^56^, ^57^, ^58^, ^59^. Studies have shown that the ANN model accurately predicts particle size for various PLNs, with polymer surfactant activity being key for PLNs^57^. Additionally, the polymer molecular weight and concentration are key for block/tri-block copolymer NPs^58^^,^^59^. Furthermore, beyond their application in small-molecule drug delivery, PLNs have emerged as promising platforms for the delivery of nucleic acids. Multiple ML methods were used to explore the effect of polymer properties on transfection efficiency for the rational design of gene vectors, including RF^60^, SVR^61^, and BO with Shapley Additive exPlanations (SHAP)‌ analysis^62^. For example, to elucidate the structure-function relationship of the poly(beta-amino ester) polymer, Gong et al.^60^ developed an RF algorithm to predict the *in vitro* DNA transfection efficiency by vectorizing its structure in a machine-readable format, achieving moderate correlation (0.57–0.66) with experimental results in RAW264.7 macrophages and Hep3B liver cancer cells.

#### 3.1.2 Natural biopolymer-based PLNs

Natural biopolymer-based PLNs represent a diverse class of delivery systems, including polysaccharide-based NPs (*e.g.*, chitosan derivatives, starch, cellulose, carrageenan, and alginate NPs), protein-based NPs (*e.g.*, gelatin NPs), and lignin NPs, etc.^63^. In the context of ML-driven formulation design of natural biopolymer-based PLNs, chitosan and its derivative-based NPs are the most extensively studied. Ionotropic gelation is the most commonly used method for their preparation *via* electrostatic interactions between chitosan and polyanions (*e.g.*, tripolyphosphate (TPP)) under acidic conditions^64^. ANN-based models demonstrated that TPP concentration had the most significant impact on both particle size and yield for chitosan-NPs^65^. Given that unmodified chitosan-NPs tend to aggregate under neutral conditions^66^, Bozuyuk et al.^67^ adopted a PEGylation strategy to modify chitosan-NPs. ANN models equipped with a single-layer Levenberg-Marquardt algorithm and a four-layer gradient descent method were selected to optimize PEGylated chitosan-NPs due to their strong predictive capabilities for size and zeta potential, respectively. Besides ionotropic gelation, ANN models were also used to optimize the formulation of chitosan-NPs prepared by polyelectrolyte complexation^68^ and ultrasonication^69^. Furthermore, Bagging was chosen as the most suitable algorithm to guide the intelligent real-time production of chitosan-NPs prepared by the flash nanoprecipitation method (FNP) *via* monitoring their size and PDI^70^. Beyond chitosan-NPs, Table 2 also enumerates various ML tools employed to guide the design and optimization for agar nanospheres^71^, fucoidan-NPs^72^, gelatin-NPs^73^, and hyaluronic acid (HA)-based NPs^74^.

Collectively, the development of PLNs for drug delivery is significantly advanced by ML techniques, which have become essential for predicting critical physicochemical properties, optimizing formulation parameters, and improving therapeutic outcomes, thereby overcoming the limitations of traditional methods. Such applications cover a wide range of polymer types and preparation techniques, and even extend to elucidating structure-function relationships in gene delivery vectors, thereby underscoring ML's versatility and effectiveness in accelerating the development of PLNs.

### 3.2 Lipid nanoparticles (LNPs)

LNPs are nanoscale vesicles formed through lipid self-assembly, featuring a uniform lipid core and a size range of 70–200 nm. With design flexibility, high payload capacity, and biocompatibility, LNPs are the gold standard for non-viral mRNA and siRNA delivery^75^. Based on a literature review, we observed that the most advanced ML techniques are predominantly applied in LNPs formulation development, likely spurred by the recent clinical success of notable LNPs such as Onpattro®, Comirnaty®, and Spikevax®^76^, ^77^, ^78^. Previous review has already explored ML applications in LNPs development, covering composition, process, characterization, and biological activity^25^, so we focus on comparing the strengths of different ML methods to offer deeper insights into LNPs formulation development.

**Figure 3:**
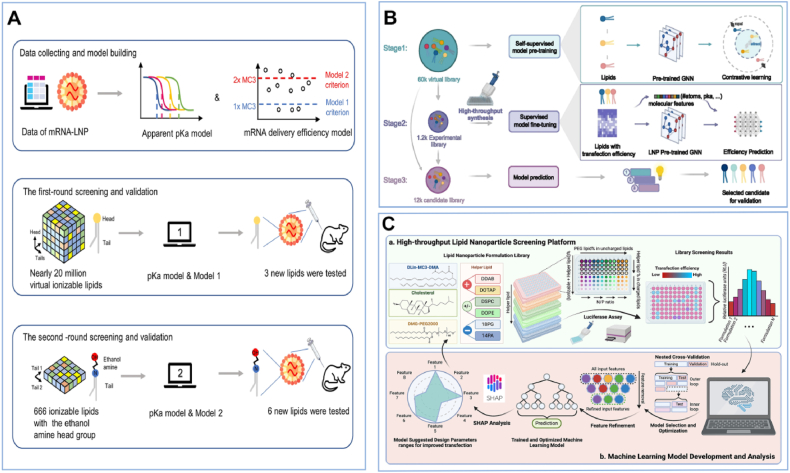
Highlights of ML-driven strategies for LNP formulation design. (A) Two round iterative AI-driven selection of ionizable lipids. Reprinted with the permission from Ref. 81. Copyright © 2024 Springer Nature. (B) Self-supervised selection of ionizable lipids *via* the AGILE platform. Reprinted with the permission from Ref. 85. Copyright © 2024 Springer Nature. (C) High-throughput screening and ML-guided selection of neutral helper lipids. Reprinted with the permission from Ref. 88. Copyright © 2024 American Chemical Society.

The formulation of LNPs is simple, with a classic composition that includes ionizable cationic lipids, PEGylated lipids, cholesterol, and neutral helper lipids^79^. Central to this system are the ionizable cationic lipids, whose unique p*K*_a_ values allow stable payload encapsulation at physiological pH and trigger endosomal release *via* protonation upon internalization^77^^,^^79^. Due to the significance of the apparent p*K*_a_ of ionizable cationic lipids^80^, Ouyang's group^81^ developed a LightGBM algorithm to predict the apparent p*K*_a_ values and mRNA delivery efficiency of literature-sourced ionizable lipids. Through two AI-driven iterative processes of generation and selection, they evaluated nearly 20 million ionizable lipids and ultimately identified six superior ionizable lipids that were experimentally validated (Fig. 3A)^81^. Given the vast structural design space for ionizable lipids, even minor alterations in their chemical properties can significantly impact the biological functions of LNPs^82^. To accelerate ionizable lipids discovery, Anderson's team^83^ combined the XGBoost algorithm with a high-throughput screening platform based on four-component reactions. To further generalize design principles across chemically diverse libraries, they employed a deep message-passing neural network to predict RNA delivery efficiency. By computationally evaluating 1.6 million lipids, they ultimately identified two ionizable lipids (*i.e.*, FO-32 and FO-35) that efficiently deliver mRNA to the lungs of mice^84^. Parallel advancements include the AGILE platform, which synergizes deep learning with combinatorial chemistry^85^. It utilizes a pretrained graph neural network to analyze millions of unlabeled lipid structures, which are refined *via* laboratory-generated transfection data (Fig. 3B)^85^. This self-supervised framework maps ionizable lipid molecular features to mRNA delivery performance while revealing cell-type-specific lipid preferences, enabling tailored LNP designs for targeted tissues^85^. Inspired by AGILE's emphasis on pretraining, Wu et al.^86^ developed TransLNP, a transformer-based model that employed pretraining and fine-tuning strategies to screen ionizable lipids. Unlike other studies, they introduced the BalMol module in the framework by balancing the similarity between adjacent targets to mitigate data imbalance. Consequently, TransLNP reduced the MSE value to 1.47 on the AGILE dataset.

Significant progress has been made on ionizable lipids, but the roles of other LNP components (*e.g.*, PEGylated lipids, cholesterol) and their complex interactions are still poorly understood, hindering rational LNP design. Evidence suggests these components also impact mRNA transfection efficiency^87^^,^^88^. For instance, Bae et al.^87^ utilized an RF algorithm to explore the impact of the type of ionizable lipids, molar ratios of ionizable lipids/neutral helper lipids (DOPE, DSPC)/cholesterol, and N/P ratio on mRNA transfection efficiency. Feature importance analysis revealed that, in addition to ionizable lipids, the molar ratio of the saturated phospholipid DSPC also plays a critical role in mRNA transfection efficiency. By integrating a high-throughput screening platform with ML algorithms, Cheng and colleagues^88^ systematically evaluated the impact of chemical structure and molar percentage of neutral helper lipids on gene transfection efficiency across six cell lines (Fig. 3C)^88^. Findings revealed that lightGBM showed more accurate prediction than RF algorithm, notably for B16F10, HepG2, and PC3 cells as evidenced by low MAE values (<0.06). Most importantly, they determined that fine-tuning helper lipid parameters is necessary for achieving cell-type-preferential transfection.

The versatility of LNPs also stems from their tunable surface properties. To enhance mRNA delivery to microglial subtypes, Rafiei et al.^89^ constructed HA-conjugated LNPs to enhance mRNA therapy for neuroinflammatory disorders. Four supervised ML classifiers (*i.e.*, RF, SVM, MLP, naïve Bayes) were employed to predict the impact of the design parameters on transfection efficiency and microglial phenotypic polarization. Among them, MLP demonstrated the best performance, with an F1-score of ≥0.8. Subsequent SHAP analysis highlighted the significance of HA modification in improving transfection efficiency and driving microglia towards an anti-inflammatory state. The transfection time of LNP-mRNA influences cellular state. Harrison et al.^90^ developed a pipeline to explore the temporal response of cells to RNA therapeutics. This pipeline utilized automated microscopy, deep learning, and gradient boosting machine models on time-series data to assess LNP-mRNA effects in HepG2 cells. Beyond using transfection efficiency as an output parameter, Ouyang's group^91^ employed LightGBM to predict the LNP formulation for mRNA vaccines based on the IgG titer. Due to LightGBM's high accuracy in classification and regression, they also used it to predict the *in vitro* and *in vivo* gene knockdown rates of siRNA-LNPs^92^.

Beyond the lipid composition and surface modifications, LNPs' transfection efficiency is also largely influenced by physicochemical parameters. Consequently, employing ML to predict physicochemical parameters (*e.g.*, size, PDI, EE, DL) is crucial for accelerating LNPs formulation development. Tree-based methods like XGBoost and RF have proven particularly effective for screening LNPs formulations^93^^,^^94^, probably because their ordered binary decision nodes enable them to more accurately identify and fit non-linear and complex patterns compared to other algorithms^88^. Furthermore, research by Jeong's group^95^ showed that a combinatorial ANN-DOE model outperformed XGBoost in predicting size, PDI, zeta potential, and EE for mRNA-LNPs with a minimum number of experiments. Optimizing LNPs formulations to meet multiple outputs simultaneously is challenging. Maharjan et al.^96^ addressed this by initially using principal component analysis (PCA) to reduce eight critical parameters. They then compared BO-optimized XGBoost against a self-validated ensemble model (SVEM) for optimizing mRNA-LNP. It was found that the SVEM model proved superior predictive accuracy (>97%) owing to its weighted bootstrapping that eliminated the need for separate training/validation datasets.

Taken together, ML has become a critical driver in optimizing and accelerating LNPs formulation development. By predicting the properties of key lipids, particularly ionizable cationic lipids, understanding complex component interactions, and optimizing physicochemical parameters, ML significantly advances the design and development of non-viral gene delivery systems.

### 3.3 Liposomes

Liposomes are spherical vesicular systems composed of phospholipids and cholesterol that self-assemble into unilamellar or multilamellar architectures^97^. Owing to their structural resemblance to natural biological membranes and tunable surface properties, liposomes have been extensively utilized for drug delivery^98^. Since the US Food and Drug Administration (FDA) first approved the liposomal product Doxil^Ⓡ^ in 1995, dozens of liposome-based formulations have been clinically approved^99^. To further advance the development of liposomes, researchers are increasingly adopting ML technologies to optimize liposomal formulations and manufacturing processes. Previous review on the application of ML in liposome development focused on the refinement of key parameters to accelerate the design and optimization process of liposome^24^. We have further summarized the detailed ML techniques utilized, as shown in Table 3^100^, ^101^, ^102^, ^103^, ^104^, ^105^, ^106^, ^107^, ^108^, ^109^, ^110^, ^111^, ^112^, ^113^, ^114^ and illustrated below.

**Table 3:** Summary of common ML tools used in liposomal formulation development.

ML tool	Input feature	Output feature	Ref.
ANN	Sonication time, hydration volume, lipid: curcumin ratio	EE,flux	100
ANN	Amount of gelatin/cholesterol	DL	101
ANN	Cholesterol/PEGylated lipid concentration, TFR, FRR	Size, PDI	102
ANN, RSM	Lipid content, ultrasound power/time	EE, lipid absolute loading	103
ANN, RSM	Pilocarpine hydrochloride, sodium deoxycholate, water content	EE	104
ANN, MLR	Amount of cholesterol/edge activator, the location of the drug into the vesicle, addition of stearylamine, type of edge activator	Size, PDI, zeta potential, EE	105
ANN, molecular descriptors	Hydrocarbon tail length, cholesterol content, osmolarity, temperature, flow rate	Size, PDI	108
ANN, SVM	TFR, aqueous: Organic volume ratio, total lipid conc.	Size, dispersity, stability	109
LSBoost	Sonication time, extrusion temperature, compositions	Size, PDI	110
LSBoost	Lipids molar ratios, particle size, sonication time, pH, PDI	EE	111
XGBoost	Amount of PEGylated lipids/cholesterol, chip geometry, FRR	Size	112
Lasso regression	Lipid composition/conc., FRR, TFR, solvent, buffer, temperature	Size, PDI	113
LightGBM, RF	Preparation time, sonication, filter diameter, preparation temperature, drug: lipid ratio, weight percent of lipid, lipid composition	Size, PDI, zeta potential, EE	114

Since ANN demonstrates exceptional capability in identifying complex patterns and establishing nonlinear relationships between independent variables and dependent outcomes, it has emerged as the most widely utilized approach in liposomal formulation design^100^, ^101^, ^102^, ^103^, ^104^, ^105^ (Table 3). For example, Azubuike's team^100^ employed ANN to investigate the critical process parameters affecting curcumin-loaded liposomes. The ANN-driven optimization method revealed that maximum EE was achieved under the following conditions: lipid/curcumin ratio = 4.35, sonication time = 15 min, and hydration volume = 25 mL. Liposomes exhibit a dual drug loading mechanism, allowing for the incorporation of hydrophobic drugs into the phospholipid bilayer and the effective encapsulation of hydrophilic therapeutics within the aqueous core^106^. However, the limited aqueous core capacity of conventional liposomes often results in low EE for hydrophilic therapeutics. To address this limitation, Metwally's team^101^ utilized an ANN to markedly improve the EE of sodium salicylate by adjusting the amounts of gelatin and cholesterol in gelatinized-core liposomes. Numerous studies have demonstrated that ANN exhibits superior predictive performance over response surface methodology (RSM)^103^^,^^104^, and MLR^105^ in liposomal formulation design and characterization. However, ANN can be limited by its restricted exploration of the factor space and tendency towards local optima, preventing deep insights into complex systems. Molecular descriptors, which numerically represent molecular properties, are well-suited for predictive modeling^107^. Sansare et al.^108^ illustrated that integrating these descriptors with ANN can more accurately predict the relationships between liposome processing parameters (*e.g.*, hydrocarbon tail length, cholesterol percentage, buffer type, solvent temperature, and flow rate) and key quality attributes (*e.g.*, particle size and PDI) of liposomes.

Conventional top-down liposome preparation methods (*e.g.*, thin film hydration, solvent injection) often result in solvent residues, high PDI, and poor reproducibility^102^. Microfluidics offers a better, bottom-up approach for uniform vesicles with improved reproducibility and lower PDI, controlled by parameters like total flow rate (TFR) and aqueous-to-organic flow rate ratio (FRR)^102^. Recent advancements have integrated ANNs with microfluidics to accelerate liposome optimization^102^^,^^109^. For example, Boso's group^109^ developed an ANN model to investigate the influence of lipid concentration, TFR, and FRR on the particle size of curcumin-loaded liposomes *via* microfluidic systems. They found TFR had the most significant impact on liposome size, followed by lipid concentration and FRR.

Besides ANN, the LSBoost algorithm also demonstrated significant advantages in predicting the relationships between liposomal formulations, manufacturing processes, and their physicochemical parameters^110^^,^^111^. For example, Eslami's team^111^ developed an LSBoost algorithm to investigate the effects of lipid molar ratios, sonication time, aqueous phase pH, particle size, and PDI on the EE of curcumin-liposome. The algorithm predicted that the maximum EE of curcumin could be achieved with an HSPC: DPPG: Chol: DSPE-mPEG2000 M ratio of 55:5:35:5, a sonication time of 30 min, a pH range of 7.2–8, a particle size of 130 nm, and a PDI range of 0.09–0.13. Furthermore, XGBoost^112^ and Lasso regression^113^ models demonstrated reliable prediction of liposome formation and size throughout a broad design space during microfluidic production. To construct a general predictive model for liposomal formulation development, Ouyang's group^114^ compiled a dataset of 665 formulations, including various lipid components and their ratios, along with corresponding particle size, PDI, zeta potential, and EE. Five algorithms were employed to identify the critical factors in liposome formulation design. Comparative analysis demonstrated that the LightGBM model exhibited the lowest MAE and the highest *R*^2^ value in predicting size and zeta potential, whereas RF demonstrated superior predictive performance in terms of PDI and EE. Feature importance analysis revealed that drug molecules exhibiting a log*S* range of −3 to −6, molecular complexity between 500 and 1000, and *X*Log*P*3 values ≥ 2 were preferred to be prepared as liposomes with high EE^114^. To facilitate global research, their group established FormulationAI, an efficient platform for predicting liposome formulations (https://formulationai.computpharm.org/)^115^.

### 3.4 Solid lipid nanoparticles (SLNs) and nanostructured lipid carriers (NLCs)

Lipid nanoparticles represent a broad category of NDDS composed of lipids. Beyond the LNPs mentioned above for mRNA/siRNA delivery, they can be classified as SLNs and NLCs based on the physical state of the lipids^116^. SLNs consist of a monolayer shell of surfactant and a solid lipid core at physiological temperature^117^. The highly ordered crystalline structure of the lipid core in SLNs imposes stringent requirements on the payload drugs. To simulate drug loading in SLNs, Hathout and coworkers^118^ developed a GP-based supervised ML algorithm to predict DL in tripalmitin-based SLNs by correlating drug descriptors with their molecular docking binding energies. To optimize the formulation and process of SLNs, multiple ML methods, including ANN, DT, Lasso, Ridge, SVM, KNN, GP, MLP, RF, Bagging, and gradient boosting, have been employed^119^^,^^120^. For instance, Öztürk et al.^120^ developed 12 ML algorithms to explore the influence of homogenization time and lipid concentration, and surfactant concentration on SLN particle size. Their study demonstrated that the DT model outperformed other algorithms in predicting particle size under different conditions. It was found that homogenization time strongly influenced particle size, with longer durations resulting in smaller particles and a moderate decrease in PDI. Lipid concentration had a weaker effect, showing a slight positive correlation with particle size.

Despite the numerous advantages of SLNs, such as enhanced oral bioavailability and biocompatibility, the highly ordered crystalline structure of the core solid lipid limits payload efficiency and might lead to drug leakage during storage^121^. To address these issues, the more advanced NLCs have been developed. As the second generation of SLNs, NLCs could improve loading capability by mixing solid lipids with liquid lipids to form an amorphous solid lipid matrix, thereby maintaining drug stability during storage^97^^,^^122^. This underscores the critical importance of lipid selection in formulation design. In a pioneering study‌, Basso et al.^116^ employed an integrated ML approach to evaluate two novel glycerol-based lipids for NLC optimization. They first used hierarchical clustering and PCA to assess structural similarity, finding that both lipids restored NLC's negative zeta potential with enhanced biocompatibility. Further ML analysis (*i.e.*, partial least squares regression and neural networks) of multiple formulation factors on NLC quality attributes (size, PDI, zeta potential, cytotoxicity, drug loading) led to successful NLC optimization^116^. Besides, to tackle the major limitation of ANN concerning the requirement for extensive training data, researchers have integrated ANN with other methodologies. Hybrids such as ANN combined with Box-Behnken design and variable weight desirability functions^123^, or ANN integrated with neuro-fuzzy logic and GA^124^^,^^125^, demonstrate great potential. These methods effectively delineated the knowledge and design spaces for NLCs, thereby enabling more systematic formulation development.

Due to the structural and functional similarities between SLNs and NLCs, researchers have increasingly leveraged ML algorithms to concurrently optimize their formulation and manufacturing process^126^, ^127^, ^128^. For instance, Bao and colleagues^128^ combined ML methods with high-throughput and miniaturized automation to speed up formulation development for cannabidiol-loaded SLNs and NLCs. In their process, automated lab equipment was first used to prepare a small subset of formulations, generating data to train eight different ML regression algorithms. They found that RF, DT, and XGBoost models were the best predictors for particle size, DL, and EE, respectively. These selected models then generated a synthetic dataset ten times larger than the initial dataset to predict optimal formulations. Impressively, these predicted formulations greatly enhanced drug solubility (up to 3000-fold), prevented degradation, and improved oral bioavailability. Collectively, ML algorithms are increasingly crucial for optimizing SLNs and NLCs. By analyzing the impact of complex factors (*e.g.*, drug properties, process parameters, and lipid composition) on NDDS characteristics (*e.g.*, particle size, EE, DL), researchers can more effectively optimize these systems, overcome their inherent limitations, and significantly enhance drug delivery performance.

### 3.5 Nanoemulsions

Nanoemulsions are colloidal dispersions (20–200 nm droplets) of oil and water stabilized by surfactants and co-emulsifiers, appearing transparent to translucent due to weak light scattering^129^. Unlike conventional emulsions, nanoemulsions exhibit both thermodynamic and kinetic stability, preventing flocculation and phase separation through steric hindrance. Their high specific surface area and permeation enhancement make them ideal for improving hydrophobic drug bioavailability^130^^,^^131^. Nanoemulsions preparation is broadly categorized into high-energy and low-energy methods. High-energy methods, like ultrasonic emulsification and high-pressure homogenization, dominate large-scale production^132^^,^^133^. ANN is the most widely adopted method in the development of nanoemulsions^130^^,^^134^, ^135^, ^136^, ^137^. Among these studies, most ANN models have found that surfactant concentration is the primary factor determining the size of nanoemulsions, regardless of whether ultrasonic emulsification^134^ or high-pressure homogenization^130^^,^^135^ was used. This could be because surfactants adsorb at the water/oil interface, requiring more surfactant for smaller droplets due to their larger specific surface area. Interestingly, Samson et al.^136^ identified xanthan gum as the key factor influencing particle size in virgin coconut oil nanoemulsion systems using a GA-enhanced ANN model. In addition to investigating the effect of formulation factors on particle size, Ngan et al.^137^ investigated the impact of process parameters such as sonication amplitude, duration, and homogenization speed on the droplet size and viscosity of nanoemulsions. To conduct this analysis, they trained an ANN model using four distinct algorithms (*i.e.*, incremental back propagation (IBP), batch back propagation (BBP), quick propagation (QP), and Levenberg-Marquardt (LM)). The results demonstrated that ultrasonic cavitation outperformed high-shear homogenization in regulating droplet size (*via* sonication time) and viscosity (*via* amplitude modulation). Additionally, the predictive capabilities of each learning algorithm ranked in the following order: LM > IBP, BBP > QP.

The clinical translation of nanoemulsions faces challenges such as limited shelf-life stability and cytotoxicity risks associated with surfactants^138^. To address these issues, Amani's team^139^ employed ANN to analyze four formulation variables (*e.g.*, the concentration of NP, oil, surfactant, and alcohol) influencing the short-term stability of superparamagnetic iron oxide-loaded nanoemulsions. The ANN model revealed that all variables exhibited a negative correlation with stability beyond their optimal thresholds. Maximum stability was achieved at specific ratios: 15.8% *v*/*v* Span 80/Tween 80, 3.6% *v*/*v* almond oil, 0.3 mg/15 cm^3^NP concentration, and 2.3% *v*/*v* ethanol. Building on this work‌, they utilized an ANN to further investigate formulation factors governing both stability and cytotoxicity in a novel nanoemulsion system. The results revealed that the most critical component for stability was the surfactant concentration, while cytotoxicity was mainly influenced by the oil concentration^139^.

### 3.6 Nanosuspensions

Nanosuspensions are colloidal drug dispersions in liquid media, offering advantages such as improved dissolution/solubility, scalable production, and enhanced stability^140^. They are generally prepared using top-down (*e.g.*, media milling, high-pressure homogenization) or bottom-up (*e.g.*, antisolvent precipitation) methods^141^. Wet media milling is one of the most commonly used top-down methods^142^, and it reduces drug particle size through collisions. However, prolonged milling often raises temperature, posing challenges for thermolabile drugs. To address this, Bilgili's group^143^^,^^144^ developed a semi-theoretical lumped parameter model enhanced with power law and KNN models to predict the temperature evolution of the solution during the preparation of nanosuspensions, providing insights for controlling heat and optimizing the process for thermolabile drugs. In contrast, the bottom-up antisolvent precipitation method creates a supersaturated solution by mixing the drug solution with anti-solvent^145^. Abdelbary and colleagues^146^ utilized this method with ultrasonication to prepare carvedilol nanosuspensions. By combining ANN with GA, they optimized the nanosuspensions based on particle size, PDI, and zeta potential, finding Pluronic F127 to be the optimal stabilizer. Similarly, ANN models were also extensively used to optimize acetaminophen and iodine (^127^I) nanosuspensions produced in microchannel reactors^147^, ^148^, ^149^. For example, Amani's team^147^ employed ANN to study acetaminophen nanosuspensions. They investigated how surfactant concentration, solvent/anti-solvent flow rates, and temperature affected sedimentation time and PDI. The results showed that anti-solvent flow rate and temperature positively influenced precipitation time, while solvent flow rate had an inverse effect. Surfactant concentration was identified as the most critical factor for stability. A deeper ANN-based analysis by the team showed that optimal PDI was achieved with higher anti-solvent flow rates and temperature, coupled with minimal solvent flow rate^148^.

Besides optimizing formulation and process parameters, ANN enables real-time quality monitoring during continuous manufacturing processes. A notable example is the work by Davidopoulou and colleagues^150^. They integrated ANN with digital twin technology to predict and optimize the production of nanosuspensions. Briefly, the ANN framework was employed for data sampling, model deployment, and dynamic simulation of milling processes, allowing precise estimation of particle size distribution at any stage of grinding. By combining ANN with digital twin technology, real-time manufacturing data can be used to dynamically adjust process parameters. This collaborative approach allows for proactive control and prediction of both process progression and the quality control of the final product^150^.

### 3.7 Lipid-based hybrid NDDS

Lipid-based hybrid NDDS refer to nanocarriers engineered by combining lipid components with other materials, such as polymers, dendrimers, or inorganic nanoparticles, to leverage the unique properties of each constituent for enhanced drug delivery performance^151^. Due to the significant potential of ML algorithms in NDDS formulation development, their application extends beyond the aforementioned lipid-based nanocarriers to hybrid lipid systems. Among various ML methods, ANN is the most widely applied. It has been utilized for the formulation optimization of posaconazole-loaded phospholipid-based nanoformulations^152^, verapamil hydrochloride-loaded polymer-lipid NPs^153^, and dendrimer-lipid nanocarriers^154^. An illustrative example is the work by Henser-Brownhill et al.^154^, who developed an ANN model for predicting mRNA delivery efficiency of dendrimer-lipid nanocarriers and RF algorithms for predicting size, PDI and zeta potential, respectively. Through the synergistic integration of these ML techniques with high-throughput screening, they created a platform capable of *in silico* evaluating millions of candidate formulations, which drastically accelerated the optimization of dendrimer lipid nanocarriers for nucleic acid delivery. Similarly, by combining the XGBoost model with high-throughput screening, Mirkin's team^155^ achieved a tenfold reduction in the number of samples requiring testing. This allowed for a more thorough exploration of the nanomedicine-design space during the development of spherical nucleic acids.

### 3.8 Self-emulsifying drug delivery systems (SMEDDS)

SMEDDS are transparent/translucent mixtures of oil, water, surfactants, and co-surfactants that spontaneously form stable and homogeneous systems without mechanical energy^156^, ^157^, ^158^. They can improve the oral bioavailability of hydrophobic drugs by increasing solubility^159^. To date, over a dozen FDA-approved products exist (*e.g.*, Gengraf®, Norvir®, Depakene®)^160^, but their development is typically slow and expensive, requiring extensive screening and optimization.

Theoretical modeling, particularly using ML algorithms, provides a powerful solution to advance the SMEDDS development. By predicting solubility, particle size, and enabling phase diagram prediction, these computational methods drastically reduce the need for extensive experimental screening and optimize the formulation process. For instance, Li et al.^161^ developed a QSPR model using MLR and ANN algorithms. This model correlates the molecular structures of oils, surfactants, cosurfactants, and 2-arylpropionic acid with drug solubility in SEDDS by encoding electronic, geometric, topological, and quantum-chemical descriptors. The resulting QSPR model allows for rapid prediction of 2-arylpropionic acid solubility in SMEDDS and quick identification of optimal formulations. Beyond solubility prediction, Parikh and coworkers^162^ constructed predictive models for the particle size of iloperidone self-emulsifying pre-concentrates (the initial physical state of SMEDDS) using I-optimal design and ANN methods. Comparative analysis demonstrated the superior predictive capability of ANN over the I-optimal design. Subsequent sensitivity analysis further revealed that oil composition exerted the strongest influence on microemulsion droplet size. Moreover, the critical technique for formulating SMEDDS involves mapping the ternary phase diagram of oil, surfactant, and co-surfactant to identify the self-emulsifying region. To enhance predictive accuracy for SMEDDS formulation properties, Ouyang's group^160^ developed a pseudo-ternary phase diagram prediction model using an RF algorithm trained on a dataset of 4495 SMEDDS formulations. They then selected meloxicam as a model drug and experimentally constructed a pseudo-ternary phase diagram for meloxicam-SMEDDS. The results indicated that the constructed prediction model performed well, achieving an accuracy of 89.51%. Furthermore, molecular dynamics simulations were employed to investigate molecular interactions between excipients and the model drug, unveiling their diffusion behaviors in aqueous environments and elucidating the mechanistic role of cosurfactants. This integrated computational approach provides novel insights into rational SMEDDS formulation design. Building on this framework, they also developed a FormulationAI platform to guide the formulation design of SMEDDS^115^.

### 3.9 Niosomes

Niosomes are closed bilayer vesicles self-assembled from non-ionic surfactants and cholesterol in water, capable of encapsulating both hydrophilic and hydrophobic drugs like liposomes^163^. Unlike liposomes, they do not require expensive phospholipids, making them more cost-effective, readily available, less prone to oxidation, and potentially lower in toxicity. Cholesterol incorporation enhances their robustness and stability. These attributes make niosomes versatile carriers for drugs and cosmetic actives^164^^,^^165^.

Given the widespread use of niosomes over the past few decades, increasing studies leverage ML algorithms to analyze historical data, establishing correlations between formulation parameters and physicochemical properties to guide their optimization^166^, ^167^, ^168^. For example, Ghaemmaghami's group^166^ developed an ML framework to predict the EE of niosomes. By collecting data from studies published over the past decade, they trained three ML models (*i.e.*, DNN, linear regression, polynomial regression) using four cost functions (MSE, MAE, MAE-M, Gaussian). The DNN model with MAE-M demonstrated the best predictive performance and identified HLB as the most influential parameter for EE. Experimental validation using seven doxycycline hyclate-loaded niosomes further confirmed the predictive accuracy of the model. Similarly‌, comparative analysis‌ by Fahmy et al.^167^ evaluated four regression algorithms (*i.e.*, categorical boosting (CatBoost), linear regression, SVR, and ANN) for EE prediction. Among them, CatBoost achieved optimal performance (*R*^2^ = 0.98, MSE<10^−4^). The subsequent feature importance analysis revealed that the drug-to-lipid ratio is the primary factor influencing EE, followed by the lipid-to-surfactant molar ratio. Expanding beyond EE prediction, Shahiwala et al.^168^ established an ANN model to predict particle size based on eleven niosome formulations and process variables, corroborating findings that drug-to-lipid and cholesterol-to-surfactant ratios are also critical determinants for size.

### 3.10 Nanocrystals

Nanocrystals are carrier-free submicron colloidal dispersions composed of pure drug active ingredients and stabilizers, exhibiting exceptionally high drug loading capacities. Their core advantage lies in the enhancement of solubility and bioavailability of poorly soluble drugs through nanoscale particle sizes. Additionally, nanocrystals offer stability, improved drug release, and scalable production^3^. They are typically produced *via* top-down (*e.g.*, milling, homogenization) or bottom-up methods (*e.g.*, antisolvent precipitation, spray drying)^169^^,^^170^. Since hydrophobic drug dissolution rate and solubility inversely depend on size, controlling particle size and PDI of nanocrystals is crucial^171^. Building on this understanding, Ouyang's group^172^ systematically collected 910 datasets on nanocrystal size and 341 datasets on PDI. To construct regression models for predicting particle size and PDI of the nanocrystals prepared by high-pressure homogenization, ball wet milling, or antisolvent precipitation, eight ML algorithms were employed, including RF, DT, LightGBM, MLR, partial least squares, KNN, SVM, and DNN. By comparing the MAE value of each algorithm, LightGBM was determined to exhibit superior predictive performance. Feature importance analysis revealed that cycle index, milling time, and stabilizer concentration are critical factors for the preparation of nanocrystals by high-pressure homogenization, ball wet milling, and antisolvent precipitation, respectively. To further validate the generalization and predictive accuracy of the constructed ML model, six novel drug nanocrystals absent in the training datasets were prepared. Experimental measurements of their particle size and PDI demonstrated strong concordance with the values predicted by the models, confirming their robustness in practical applications.

## 4 ML-aided prediction of critical quality attributes in NDDS formulation development

The safety and efficacy of NDDS are highly dependent on their critical quality attributes. Even minor deviations in these parameters can significantly alter pharmacokinetic profiles or even lead to potential safety risks^173^. In the context of ML empowered NDDS formulation development mentioned in Section 3, certain critical quality attributes emerge as particularly critical metrics, including particle size and distribution, morphological structure, surface charge, EE and DL, drug release behavior, and stability, as summarized below.

### 4.1 Prediction and characterization of particle size and distribution *via* ML

The particle size of NDDS not only influences critical parameters such as drug loading capacity and release kinetics^174^, but also profoundly impacts their pharmacokinetics, biodistribution, clearance pathways^175^, and even delivery mechanisms^176^. Additionally, the size distribution reflects the stability and uniformity of NDDS. Consequently, precise control over both particle size and distribution is essential. Recognizing this, numerous ML algorithms have been applied in predicting particle size and distribution during formulation design and optimization of diverse NDDS. Among various ML methods, ANN has the widest application, being used in almost all the development of NDDS covered in this review^46^^,^^47^^,^^53^^,^^57^, ^58^, ^59^^,^^65^^,^^67^, ^68^, ^69^^,^^73^^,^^74^^,^^102^, ^103^, ^104^, ^105^^,^^109^^,^^130^^,^^134^, ^135^, ^136^, ^137^^,^^148^, ^149^, ^150^^,^^152^^,^^162^. For scenarios with limited data or where experimental design integration is crucial, hybrid approaches combining ANN with techniques like DOE^72^^,^^95^, GA, molecular descriptors^108^, variable weight desirability functions^123^, or neuro-fuzzy logic^124^ have shown great advantages. Besides, other neural network-based algorithms (*e.g.*, neuro-fuzzy logic^52^, GCN-FCNN^54^, MLP^55^) have also been proven effective in predicting particle size and distribution. Another significant category is ensemble algorithms. These methods improve accuracy and robustness by leveraging the combined predictions of multiple models, often also reducing the risk of overfitting, making them highly recommended choices. Within this category, several ensemble algorithms have shown particularly strong predictive performance, including XGBoost, LSBoost^110^^,^^111^, lightGBM^114^^,^^172^, BAG-SVR^44^, ADA-KNN^45^, Bagging^70^, SVEM^96^, and RF. It should be noted that RF is also a typical representative of the Bagging strategy in ensemble learning. Additionally, a range of other established ML algorithms have been successfully employed for this task, such as DT^49^^,^^120^, SVR^50^, and Lasso regression^113^.

Furthermore, ML algorithms have been applied in the accurate characterization of these parameters. Although particle size and distribution data of NDDS are typically obtained using the dynamic light scattering method, microscopic imaging techniques (*e.g.*, transmission electron microscopy, atomic force microscopy) offer alternative means to visualize and analyze particles^177^. Recently, neural network-based image processing algorithms have been used for determining NDDS sizes^16^, ^17^, ^18^ and dispersity^18^.

### 4.2 Morphological structure characterization *via* ML

The morphological structural attributes of NDDS are fundamental determinants of their biological behavior, critically influencing interactions with biomolecules (*e.g.*, proteins and cell membranes), drug release profiles, and intracellular trafficking pathways^178^. Given this profound impact on performance, the morphological structure emerges as an output parameter in NDDS formulation development *via* computational methods^19^. Nanotechnologies yield diverse architectures, including solid nanoparticles, hollow nanocapsules, core–shell structures, or multilayer structures with common morphologies encompassing spherical, quasi-spherical, rod-like, or fibrous geometries^1^. These morphological features are typically assessed using advanced imaging methodologies. Recently, the majority of studies have utilized deep learning approaches to analyze microscopic images of NDDS for the purpose of identifying their morphologies^19^, ^20^, ^21^, ^22^, ^23^. Among them, many of these approaches rely on supervised learning, which requires substantial manual effort for data annotation. To circumvent these constraints, *K*-means clustering and Naïve Bayes classifier-based unsupervised ML algorithms have been engineered to autonomously extract intrinsic geometric properties of NDDS with minimal manual input, yielding substantially improved analytical reproducibility and throughput^19^.

### 4.3 Surface charge prediction *via* ML

The surface charge of NDDS is a critical determinant of their behavior in biological systems, profoundly influencing aggregation propensity, colloidal stability, cellular uptake, and biodistribution patterns^179^^,^^180^. This makes it a particularly significant output parameter in ML-driven NDDS formulation development and quality control. Although surface charge itself is influenced by factors such as material composition and the dispersion medium, measuring its zeta potential yields a crucial and quantifiable metric. Consequently, accurately predicting and controlling zeta potential is vital. ANN has become widely adopted for this purpose, successfully assessing zeta potential across diverse NDDS formulations, including polymethacrylate-based NPs^53^, chitosan-based NPs^65^^,^^67^, agar nanospheres^71^, timolol maleate-loaded liposome^105^, and mRNA LNPs^95^. Beyond conventional ANN frameworks, ensemble algorithms (such as lightGBM^114^, XGBoost^43^^,^^93^^,^^94^, RF, BAG-PR^44^, and SBNNR^45^) are often preferred because they can integrate the advantages of different models, typically providing more accurate and robust predictions, making them a worthy option for prioritization.

### 4.4 Drug encapsulation efficiency (EE) and drug loading efficiency (DL) predictions *via* ML

EE enhances therapeutic stability under physiological conditions and enables controlled release kinetics. Optimal DL further ensures clinical efficacy aligned with dosing regimens^181^. EE and DL are closely related to the formulation composition and preparation process of NDDS. Alongside size and PDI, EE and DL are the most common output parameters for NDDS optimization^50^^,^^51^^,^^72^^,^^105^. To streamline the design and optimization of diverse NDDS, ML algorithms have been widely used to predict EE and DL. This approach mirrors the application of ML in predicting NDDS size and PDI, where ANN^46^^,^^58^^,^^68^^,^^73^^,^^100^^,^^101^^,^^103^, ^104^, ^105^ and their hybrid models^71^^,^^72^^,^^95^^,^^96^^,^^123^ were predominantly employed. Ensemble algorithms, such as RF, XGBoost^93^^,^^94^, LSBoost^111^, CatBoost^167^, and SVEM^96^, also demonstrated superior predictive performance. Moreover, the study also found that GP^48^^,^^51^, linear regression^50^, and DNN^166^ have been proven effective for EE and DL predictions.

Besides optimizing formulation and process parameters^100^^,^^101^^,^^103^^,^^104^^,^^111^, ML methodologies demonstrate unique capabilities in screening drug candidates with superior encapsulation potential for various NDDS *via* AI-driven QSPR models, such as liposomes^14^^,^^182^ and polymeric micelles^15^^,^^183^^,^^184^. Furthermore, due to the complexity of directly measuring EE and DL, a GP-based framework was established to predict EE and DL indirectly by generating predictive maps based on particle size data. Such maps facilitate the rapid identification of optimized PLGA-NPs, thereby significantly reducing reliance on trial-and-error experimentation^51^. Moreover, it has been reported that GP-based supervised ML algorithms have also been applied to predict DL of any drug in the studied tripalmitin-based SLNs by correlating drug descriptors with their molecular docking binding energies^118^.

### 4.5 Drug release behavior prediction *via* ML

*In vitro* drug release behavior serves as a critical quality control indicator for NDDS and provides valuable insights into their potential *in vivo* performance. This performance can profoundly influence absorption, *in vivo* safety and efficacy profiles, and even the long-term stability of the formulation^185^^,^^186^. To better understand the underlying release mechanisms, several mathematical models have been developed to describe drug release behavior, such as zero-order, first-order, Hixson-Crowell, Higuchi, Weibull, Gompertz, and Korsmeyer-Peppas models^187^. However, these empirical models are primarily useful for post hoc analysis of experimental data and lack the predictive power necessary for guiding formulation design.

To gain deeper insights into drug release dynamics and enhance predictive capabilities, researchers are increasingly adopting ML empowered approaches^188^. In the context of PLNs, various algorithms have been utilized to forecast *in vitro* release behavior, such as ANN^56^^,^^189^, GA-ANN^71^^,^^190^, GP^191^, and LightGBM^190^, ^191^, ^192^. Among these research studies, Allen's group^192^ conducted a systematic investigation comparing the performance of ten ML algorithms for predicting the release kinetics of polymer-based long-acting injectables. The results showed that tree-based models (*e.g.*, DT, RF, XGBoost, natural gradient boosting, LightGBM) generally achieved higher prediction accuracy than linear (*e.g.*, partial least squares, Lasso, MLR), instance-based (*e.g.*, KNN), and deep learning models (*e.g.*, neural network). Specifically, LightGBM demonstrated the highest predictive accuracy^190^, ^191^, ^192^. Nevertheless, the limited quantity and heterogeneity of available data often constrain the predictive accuracy of these ML models. To address this, Bannigan et al.^192^ developed a larger dataset containing 181 drug release profiles with 3783 individual fractional release measurements for 43 unique drug-polymer combinations. Their analysis also confirmed LightGBM as optimal for predicting drug release from PLGA, PLA, or PCL-based polymeric systems. Furthermore, beyond direct prediction, ML algorithms (*e.g.*, neural network, gradient boosting regression, DT, extra Tree, ANN) can be used to estimate key parameters like diffusion coefficients in erosion models^193^, drug concentration within the polymeric matrix at various locations^194^, ^195^, ^196^, and partition coefficients^197^, enabling indirect drug release prediction from PLNs.

Beyond predicting drug release from polymeric NPs, some ML methods have been applied to predict the *in vitro* release behavior of liposomes, including deep learning^198^, ANN^199^^,^^200^, and RF^201^. These models can forecast drug release profiles not only under simulated physiological conditions but also under varying pH environments^198^ and different ultrasound frequencies^200^^,^^201^. Furthermore, predictive models have been successfully constructed for diverse carriers such as lipidic mesophase drug carriers^202^, lecithin/chitosan NP^203^, and PEGylated KIT-6 NP^204^, utilizing techniques like XGBoost, DNN, and non-linear generalized ANN, respectively.

### 4.6 Stability predication *via* ML

Nanoparticle aggregation poses a significant challenge to the stability of NDDS. ML algorithms have been successfully applied to classify the aggregation states of these systems. For instance, the SVM model was proven effective in distinguishing the stable and unstable states of liposomes based on PDI data^109^. ANN has been employed to predict the stability of nanoemulsions through transparency measurements^139^^,^^205^. Beyond aggregation, drug leakage during storage is another critical destabilization mechanism in NDDS, significantly impacting both shelf-life and the integrity of the drug upon administration. To tackle this issue, a QSPR model established by iterative stochastic elimination and leave-one-out validation schemes can specifically target drug leakage from liposomes during storage^206^.

Taken together, ML significantly aids scientists in accurately predicting key characteristics of nanomedicines, thereby accelerating the development of safer and more effective drugs. The optimal ML algorithm selection depends on the specific task and data availability, with ensemble algorithms like LightGBM, XGBoost, and RF excelling in high-accuracy predictions for highly non-linear problems with sufficient data, while hybrid ANN models incorporating DOE or GA are more suitable for limited data or integrated optimization. Although deep learning offers potential for image analysis, it requires extensive labeling, making unsupervised methods more practical for rapid analysis. Moreover, specialized algorithms like GP have unique advantages in predicting EE/DL through indirect parameters or screening drugs with high encapsulation potential, making them suitable for specific scenarios.

## 5 Conclusions, challenges and perspectives

The rapid advancement of AI, particularly ML techniques, is profoundly transforming research paradigms across multiple disciplines. In the development of NDDS, ML technology demonstrates substantial potential through its robust data analytics and predictive capabilities. Unlike the traditional, experience-dependent, and laborious trial-and-error development process, ML can efficiently analyze vast datasets. It not only aids in the design, optimization, and characterization of NDDS, but also shows potential for assisting in quality control, thereby remarkably accelerating the NDDS research pipeline. However, despite these significant advantages, the application of ML in NDDS development still faces several critical challenges.

The primary challenges hindering the application of ML in NDDS development are data insufficiency, fragmentation, and multimodality. The development of NDDS is highly complex, requiring substantial time and cost to obtain huge amounts of high-quality experimental data^207^. Besides, inconsistencies in technical standards and characterization methods, coupled with differing research focuses across disciplines, contribute to data fragmentation and imbalance. Taking NDDS size and distribution analysis as an example, some labs use the dynamic light scattering method, while others use transmission electron microscopy. However, regardless of the method used, the data are difficult to compare directly, which makes it hard to establish a unified ML model across studies. Apart from that, the inherent complexity of NDDS development, encompassing formulation design alongside systematic investigation of physicochemical properties, drug release kinetics, *in vitro* and *in vivo* efficacy, and safety profiles, generates multi-modal data. These data-related issues frequently lead to ML models suffering from overfitting or underfitting, resulting in a lack of robustness and generalizability^28^.

Moreover, most ML models act as “black boxes”, lacking interpretability, making it hard to understand the internal logic and basis for their predictions^208^. This might hinder regulatory approval and erode trust among healthcare professionals and patients^209^. For instance, consider an ML model that suggests a particular polymer-drug ratio for a PLN formulation based on its training data. Without interpretability features, researchers cannot determine the rationale behind this recommendation. This absence of transparency makes it hard to convince regulators of the model's reliability and complicates troubleshooting if the formulation subsequently runs into problems.

Additionally, ethical and safety concerns arise, including potential algorithmic bias, data privacy, and the potential safety and efficacy risks of ML-designed nanomedicines in clinical settings^210^^,^^211^. For example, if historical experimental data used to train an ML model primarily comes from male subjects, the model might exhibit bias when predicting the efficacy or toxicity of a nanomedicine in female subjects, potentially leading to inappropriate clinical decisions.

Despite these hurdles, promising trends are shaping the future of ML in NDDS formulation development. Addressing the data bottleneck, researchers are increasingly extracting data from publications, utilizing open databases, and leveraging high-throughput computing and experimentation to expand data availability. Concurrently, the establishment of open nanomaterial databases and sharing platforms represents a significant advancement, enabling researchers to access and utilize standardized, high-quality data more readily^212^. Building on this enhanced data foundation, the integration of multi-omics data (*e.g.*, genomics, proteomics, metabolomics) holds immense promise for personalized nanomedicine^213^.

To tackle algorithmic challenges, selecting methods appropriate for the specific datasets is crucial. Techniques suitable for small sample sizes, such as data augmentation, can be advantageous^49^. Careful comparison of different ML algorithms’ predictive performance, coupled with advanced preprocessing^45^, algorithmic optimization^71^^,^^190^, and rigorous validation techniques like *K*-fold cross-validation, leave-one-out, and hold-out validation, can mitigate the risk of overfitting, especially on small datasets^214^^,^^215^. Besides, hybrid approaches that combine ML algorithms with established techniques like DOE^95^, combinatorial chemistry^85^, or high-throughput screening^83^^,^^154^ also offer potential benefits.

To enhance the interpretability of ML algorithms, the focus on Explainable AI (XAI) is increasing^216^. This shift involves moving beyond basic tools like SHAP and Local Interpretable Model-agnostic Explanations towards more sophisticated methods that offer deeper insights into model decision-making^217^^,^^218^. Such enhanced transparency is vital not only for scientific understanding but also for gaining trust from regulatory bodies and clinicians^209^. Furthermore, addressing data privacy concerns, particularly with sensitive NDDS data, federated learning provides a valuable solution. It allows ML models to be trained across institutions without sharing raw patient data, facilitating large-scale collaborations while preserving data security^219^. Other techniques, including multi-task learning, active learning, data synthesis, transfer learning, and one-shot learning, also contribute by improving the utilization of constrained or sensitive data while maintaining privacy and boosting model utility^220^.

Furthermore, collaborations between academia, industry, and regulatory bodies are also essential to address ethical and privacy concerns. Joint workshops, pilot projects, and the development of shared ethical guidelines can facilitate dialogue and cooperation^33^^,^^221^. For instance, regulatory agencies like the NMPA or FDA could collaborate with academic researchers and industry partners to establish standardized evaluation protocols for ML-designed NDDS development. Crucially, the path from ML-optimized formulation to successful clinical translation is fraught with its own set of challenges. Key hurdles include scaling from lab to clinical manufacturing, maintaining quality control, navigating regulations requiring safety/efficacy data, and the fact that predictive models must be validated by *in vivo* tests and human trials to confirm performance and safety. Overcoming these requires interdisciplinary teamwork and a careful, validation-driven process. Close collaboration between computational scientists, experimental biologists, clinicians, and regulatory experts is vital to bridge the gap between promising ML predictions and successful clinical outcomes.

In summary, ML shows great promise for NDDS development. Due to potential hurdles like data acquisition, interpretability, ethics, and regulation, proactive approaches such as active data integration, algorithm optimization, explainable AI, and cross-disciplinary and institutional collaboration are highly encouraged to accelerate formulation development and advance clinical translation of nanomedicine.

## Author contributions

Chunyan Shen and Mengyan Zhang conducted the literature review, data collection and wrote the manuscript. Meiting Lu, Errong Chang and Ziting Gao participated part of the literature review and data collection. Weikang Ban performed the drawing and part of the literature review. Qiang Liu revised the manuscript. Zhong Zuo and Cuiping Jiang designed the research and revised the manuscript. All of the authors have read and approved the final manuscript.

## Conflicts of interest

The authors have no conflicts of interest to declare.
